# Exclusive Enteral Nutrition Plus Immediate vs. Delayed Washed Microbiota Transplantation in Crohn's Disease With Malnutrition: A Randomized Pilot Study

**DOI:** 10.3389/fmed.2021.666062

**Published:** 2021-10-22

**Authors:** Liyuan Xiang, Yan Yu, Xiao Ding, Hui Zhang, Quan Wen, Bota Cui, Faming Zhang

**Affiliations:** ^1^Medical Center for Digestive Diseases, Second Affiliated Hospital of Nanjing Medical University, Nanjing, China; ^2^Key Lab of Holistic Integrative Enterology, Nanjing Medical University, Nanjing, China; ^3^Department of Gastroenterology, Changshu No. 2 People's Hospital, Suzhou, China; ^4^Division of Clinical Nutrition, Second Affiliated Hospital of Nanjing Medical University, Nanjing, China; ^5^Division of Microbiotherapy, Sir Run Run Hospital, Nanjing Medical University, Nanjing, China; ^6^National Clinical Research Center for Digestive Diseases, Xi'an, China

**Keywords:** fecal microbiota transplant, washed microbiota transplantation, Crohn's disease, exclusive enteral nutrition, malnutrition, safety

## Abstract

**Background:** The potential of washed microbiota transplantation (WMT) in Crohn's disease (CD) has been reported. This study aimed to explore the suitable timing of WMT in patients with CD complicated with malnutrition.

**Methods:** This is a randomized, open-label study. Patients with active CD complicated with malnutrition were included and 1:1 randomized to undergo WMT at day 1 (group WMT-DAY1) or day 8 (group WMT-DAY8). The observation duration was 15 days. Exclusive enteral nutrition (EEN) was administered in both groups. The primary outcome was the improvement in nutritional parameters at day 8 and day 15 in two groups. The secondary outcome was the rate of clinical remission at day 15 in two groups.

**Results:** Totally 19 patients completed the trial. At day 8, the lymphocyte count, albumin and prealbumin increased significantly compared to those at day 1 in group WMT-DAY1 (*p* = 0.018, *p* = 0.028, *p* = 0.028, respectively), while no significant increase in any nutritional parameter was shown in group WMT-DAY8. At day 15, albumin increased significantly compared to that at day 1 in both groups (*p* < 0.05), while significant increase in prealbumin was only shown in group WMT-DAY1 (*p* = 0.004) compared to that at day 1. The rate of clinical remission at day 15 in group WMT-DAY1 and group WMT-DAY8 was 87.5% (7/8) and 72.7% (8/11), respectively (*p* = 0.603).

**Conclusion:** EEN combined with immediate WMT intervention could rapidly improve the nutritional status and induce clinical remission in malnourished patients with CD.

**Clinical Trial Registration:** www.ClinicalTrials.gov, identifier: NCT02897661.

## Introduction

The prevalence of malnutrition in patients with Crohn's disease (CD) ranged from 16–85% through reports (1, 2). Dietary restriction, disease activity and history of abdominal surgery are associated with higher risk of malnutrition (2). Exclusive enteral nutrition (EEN) is an effective treatment to improve nutritional status and induce remission in patients with CD, especially for pediatric patients (3). It is hypothesized to control disease activity by leading to profound alterations in the intestinal microbiota, enhancing barrier function and promoting direct anti-inflammatory effects (3). However, a reduction in microbiota diversity was the most frequently reported effect of EEN according to a systematic review despite variations of bacterial shifts, diversity alterations and metabolomics changes among studies (4). Therefore, there raised a clinical question that whether EEN combined with microbiota transplantation can bring much more benefits to those malnourished patients with CD. Fecal microbiota transplantation (FMT) is an effective way of remodeling gut microbiota. Our previous studies and Sokol et al., have demonstrated the therapeutic potential of FMT in inducing clinical remission and improving nutritional status in CD (5–8).

The intestinal barrier injury in CD reasonably raises the safety concern about FMT. The improved methodology of FMT in our group since 2014 was different from the traditional manual FMT and was named as washed microbiota transplantation (WMT) by the recent consensus statement (9), which was based on the automatic facilities and washing process (9, 10). The rate of WMT related adverse events (since April 2014) was 8.7% in patients with CD, which was significantly lower than 21.7% in those who underwent manual FMT from 2012 to April 2014 (11). The improved safety from FMT to WMT provided the technical support for the clinical decision on delivering microbiota suspension into intestine in patients with intestinal barrier injury.

Therefore, another raised critical question is that when is the proper time to combine WMT for those patients with CD requiring EEN. This study aimed to answer this question based on a randomized trial. The results will provide clinical evidence on using EEN and WMT in proper time for malnourished patients with CD needing comprehensive treatment strategy.

## Methods

### Study Design

This is a randomized, prospective, open-label, single-center clinical trial (NCT02897661) conducted at the Second Affiliated Hospital of Nanjing Medical University between August 2016 and May 2019.

Inclusion criteria included: patients aged 18–65 years with active CD, as defined by Harvey-Bradshaw Index (HBI) score > 4; patients with malnutrition as assessed by Nutritional Risk Screening 2002 (NRS2002) score ≥ 3 (12) and Patient-Generated Subjective Global Assessment (PG-SGA) score ≥ 4 (13). The exclusion criteria were as follows: patients complicated with contraindications of enteral nutrition (EN) such as ileus, active gastrointestinal bleeding, and shock; severe comorbidities (e.g., *Clostridioides difficile* infection, diabetes, cancer, cardiopulmonary failure and severe liver and kidney diseases); parenteral infection such as urinary infection, pneumonia, etc.; intestinal fibrotic stenosis; steroids or biologics use in 6 weeks before WMT; EN in progress; mental disorders. Written informed consents were obtained from all subjects before they participated in the study. The study was conducted in accordance with the Declaration of Helsinki and the protocol was approved by the Institutional Ethical Review Board of the Second Affiliated Hospital of Nanjing Medical University (2015LPIIT00301).

The duration of this trial was 15 days. Patients were 1:1 randomized to immediate WMT intervention group (underwent WMT at day 1, group WMT-DAY1) and delayed WMT intervention group (underwent WMT at day 8, group WMT-DAY8) using a computer-generated permuted block randomization (block sizes four). The sequence of randomization was contained in sealed opaque envelopes and kept by the clinical research coordinator. The attending doctors were responsible for enrolling patients. Patients were supplied with energy of 30–35 kcal/kg per day. EN was administered during the whole 15 days through nasogastric tube or mid-gut transendoscopic enteral tube (TET) (14) connected to a peristaltic pump. Parenteral nutrition was added properly to meet the calculated energy requirements. EEN was performed when EN provided at least 60% of targeted energy requirements (usually within 2–3 days after initiating EN). Patients were only allowed to have water but no other food through mouth. Patients were suggested to maintain their medications during the trial.

Demographic information of each participant was evaluated at baseline. Clinical activity and laboratory parameters were assessed and recorded, respectively at baseline, day 8, and day 15 of the observation period, which included HBI, hematocrit, platelet, C-reactive protein (CRP), total protein, and nutritional parameters (hemoglobin, lymphocyte count, albumin and prealbumin). Clinical remission was defined by HBI score ≤ 4. The primary outcome was the improvement in nutritional parameters at day 8 and day 15 in two groups. The secondary outcome was the rate of clinical remission at day 15 in two groups. The study was designed as a pilot trial; therefore, no sample size calculation was performed.

### Donors and WMT Procedure

Donors were screened according to our previously reported criteria (15). Healthy donors aged 21–26 years were selected from our universal stool bank (Chinese fmtBank). The improved methodology of FMT based on the automatic facilities and washing process was named as WMT. Fecal microbiota was enriched using an automatic purification machine (GenFMTer, Nanjing, China). The obtained suspension was then transferred for centrifugation at a speed of 3,000 rpm for 3 min and the supernatant was discarded. This procedure was repeated three times using sterile saline to make the suspension. The final sediment was suspended again with sterile saline in accordance with the ratio of sediment to saline (1:2). We adopted the one-hour WMT protocol in which the process time from defecation to the infusion of fresh bacterial material into patient's intestine was within 1 h (16, 17). The fresh microbiota suspension could be infused into the distal duodenum of patients through gastroscope under anesthesia. In order to prevent the reflux of microbiota suspension and inhibit the secretion of gastric acid, patients were given intramuscular metoclopramide 10 mg and intravenous proton pump inhibitor (PPI) at least 1 h before WMT (5). Another way to deliver the fresh microbiota suspension into the mid-gut was through the mid-gut TET tube (FMT Medical, Nanjing, China) (14) without metoclopramide and PPI use.

### Safety

Adverse events (AEs) were recorded in 1 month following WMT. The Common Terminology Criteria for Adverse Events (version 5.0) were used to describe the intensity and relationship of the adverse events with WMT. The intensity of AEs was graded as mild (grade 1), moderate (grade 2), severe (grade 3), life threatening (grade 4), and death (grade 5). Relativity between AEs and WMT was categorized as definitely related, probably related, possibly related, and unrelated (18).

### Statistical Analysis

According to the distribution, either paired *t*-tests or non-parametric, two-tailed, matched-pairs Wilcoxon signed-rank tests were performed to identify the differences in clinical parameters between day 1 and day 8 or between day 1 and day 15 within each group. Unpaired *t*-tests or non-parametric Mann–Whitney *U*-tests were used to assess the differences in clinical parameters between day 1 and day 8 or day 1 and day 15 in the group WMT-DAY1 vs. group WMT-DAY8. The rate of clinical remission and AEs were compared between groups using Fisher's exact test. A two-tailed *P*-value < 0.050 was considered significant. Statistical analysis was performed using IBM SPSS Statistics version 20.0 (SPSS Inc., Chicago, IL, USA). Graphics were drawn with the Prism software v.8.0 (GraphPad Software).

## Results

### Patient Characteristics

**Figure 1:**
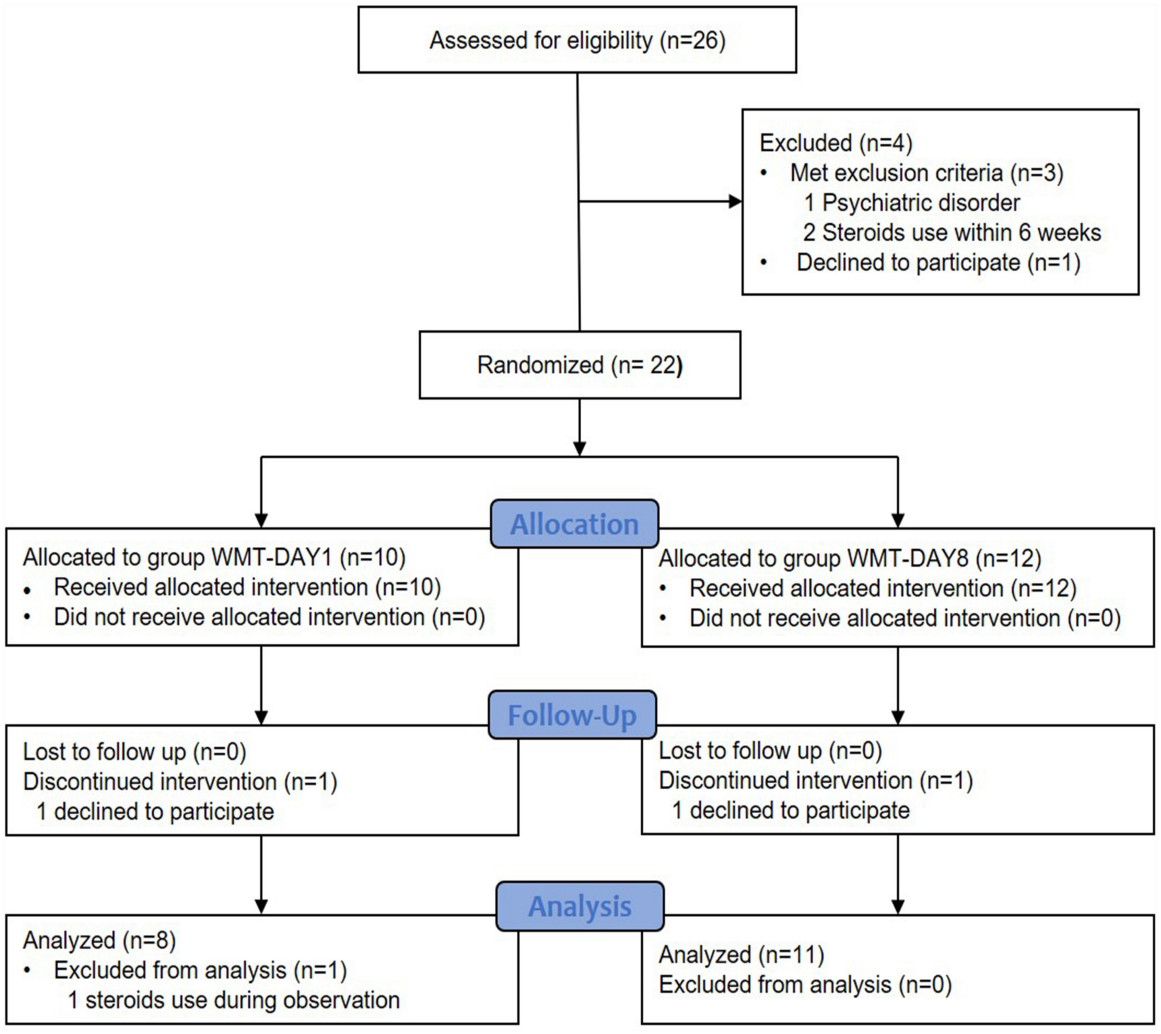
Flow of patients in the trial. WMT, washed microbiota transplantation.

In total, 26 patients were considered for enrollment between August 2016 and May 2019. Four patients were excluded: met exclusion criteria (*n* = 3) and declined to participate (*n* = 1). Twenty-two patients were randomly assigned to group WMT-DAY1 and group WMT-DAY8. Finally, 8 patients in group WMT-DAY1 and 11 patients in group WMT-DAY8 were included into analysis (Figure 1). The patient's characteristics are presented in Table 1. There were no differences in sex, age, disease duration, HBI and nutritional status between two groups at baseline.

**Table 1:** Baseline demographic and clinical characteristics of the patients.

**Item**	**WMT-DAY1**	**WMT-DAY8**	***p*** **-value**
Sex, male, *n* (%)	3 (37.5)	8 (72.7)	0.181
Age, year (median; IQR)	22 (20-54)	31 (27-37)	0.545
Disease duration, year (median; IQR)	6 (2-8)	4 (1-5)	0.395
Disease location (L1/L2/L3/L4)	0/3/5/0	1/2/8/2	0.564
Behavior (B1/B2/B3)	2/3/3	3/4/4	1.000
Perianal disease, *n* (%)	3 (37.5)	6 (54.5)	0.650
Hemoglobin, g/L	111.9 ± 13.2	106.8 ± 22.3	0.575
Hematocrit (%)	34.6 ± 3.6	33.7 ± 5.7	0.724
Platelet (10^9^/L)	334.8 ± 91.5	369.3 ± 149.5	0.572
Lymphocyte count (10^9^/L)	1.4 ± 0.6	1.3 ± 0.4	0.756
CRP	34.6 ± 18.7	15.2 ± 14.7	0.021
Total protein, g/L	70.3 ± 10.3	63.7 ± 8.2	0.129
Albumin, g/L	33.8 ± 4.6	34.5 ± 5.7	0.801
Prealbumin, mg/dl	14.2 ± 6.2	18.5 ± 5.3	0.125
BMI, kg/m^2^	19.0 ± 2.0	17.9 ± 2.1	0.091
Weight, kg	51.6 ± 4.3	51.4 ± 9.2	0.950
Harvey-Bradshaw Index	5.8 ± 0.9	7.0 ± 2.0	0.206

*WMT, washed microbiota transplantation; IQR, interquartile range; CRP, C-reactive protein; BMI, body mass index*.

### Changes in Nutritional and Inflammatory Parameters

**Figure 2:**
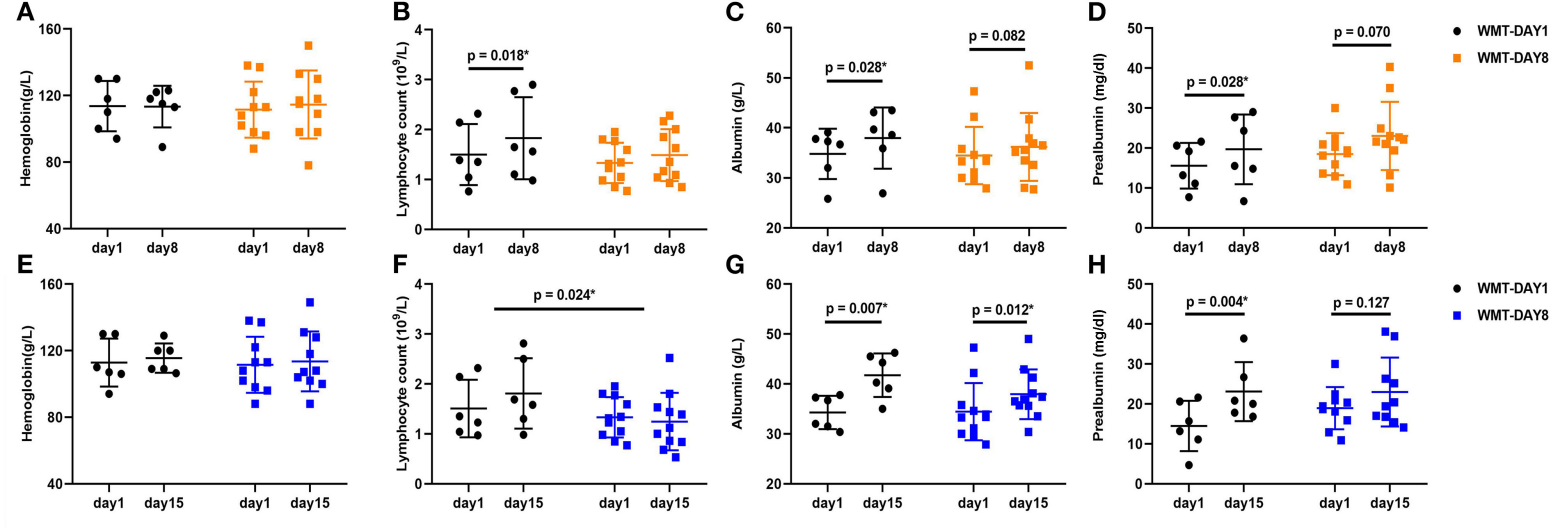
Changes in nutritional parameters between day 1 and day 8, day 1 and day 15 in two groups. **(A–D)** Changes in hemoglobin, lymphocyte count, albumin, and prealbumin between day 1 and day 8 in group WMT-DAY1 and group WMT-DAY8, respectively. **(E–H)** Changes in hemoglobin, lymphocyte count, albumin, and prealbumin between day 1 and day 15 in group WMT-DAY1 and group WMT-DAY8, respectively. The distribution of values within each group at each timing is illustrated by mean and SD. In group WMT-DAY1, data of two patients at day 8 and another two patients at day 15 were unavailable. WMT, washed microbiota transplantation.

At the first observation point (day 8), the lymphocyte count, albumin and prealbumin increased significantly compared to those at day 1 in group WMT-DAY1 (*p* = 0.018, *p* = 0.028, *p* = 0.028, respectively), while no significant difference in any nutritional parameter was shown in group WMT-DAY8. At the second observation point (day 15), the albumin and prealbumin increased significantly compared to those at day 1 in group WMT-DAY1 (*p* = 0.007, *p* = 0.004, respectively), and only albumin increased significantly in group WMT-DAY8 (*p* = 0.012). There was no significant difference in changes of nutritional parameters from day 1 to day 8 between group WMT-DAY1 and group WMT-DAY8. However, the increase in lymphocyte count from day 1 to day 15 was significantly higher in group WMT-DAY1 when comparing with group WMT-DAY8 (Figure 2). The body weight in both groups tended to increase at day 15 compared to that at day 1 but no statistical significance was detected. CRP tended to decrease at day 8 when comparing with that at day 1 in both groups (*p* = 0.116 in group WMT-DAY1 vs. *p* = 0.075 in group WMT-DAY8) (Supplementary Figure 1).

### Changes in Clinical Activity

At day 8, 87.5% (7/8) and 63.6% (7/11) of patients achieved clinical remission in group WMT-DAY1 and group WMT-DAY8, respectively (*p* = 0.338). At day 15, 87.5% (7/8) and 72.7% (8/11) of patients achieved clinical remission in group WMT-DAY1 and group WMT-DAY8, respectively (*p* = 0.603).

### Safety

WMT-related AEs occurred in 21.1% (4/19) of patients within 1 month after WMT, among which one occurred in group WMT-DAY8 and three occurred in group WMT-DAY1 (9.1 vs. 37.5%, *p* = 0.262). Of these four AEs (shown in Table 2), three occurred within 1–6 h after WMT and were transient and relieved without medical intervention. Pericoronitis was reported 2 weeks after WMT in a patient with history of poor tooth cleaning habits, which was characterized by the pain and infection of the gum tissue surrounding a partially erupted third molar. No serious AE was observed.

**Table 2:** Washed microbiota transplantation (WMT)-related adverse events (AEs) during the 1 month follow-up.

**Cases**	**AEs**	**Group**	**Duration**	**Grade**	**Causality between AEs and WMT**	**Clinical treatment and outcome**
1	Abdominal pain	WMT-DAY8	1–6h	1	Probable	Self-improvement
2	Abdominal pain	WMT-DAY1	1–6h	1	Probable	Self-improvement
3	Increased stool frequency	WMT-DAY1	1–6h	1	Probable	Self-improvement
4	Pericoronitis	WMT-DAY1	2 weeks	1	Possible	Self-improvement

## Discussion

The principal finding of this pilot study was that EEN combined with immediate WMT intervention brought a more rapid improvement in nutritional status in patients with CD compared with delayed WMT intervention, as patients in group WMT-DAY1 showed more significant increase in nutritional parameters at day 8 and day 15.

The strategy of using WMT plus EEN to treat patients with CD has been reported as part of step-up FMT strategy and showed efficacy in CD-related clinical targets including abdominal pain, hematochezia, fever, diarrhea and enterocutaneous fistula (7, 19). We considered that the efficacy of this strategy in improvement of nutritional status and disease activity should be better than using WMT or EEN solely.

Evidence showed that transferring diet-responsive bacterial taxa between hosts could enhance subsequent responses to diet interventions (20), which indicated that microbial composition may affect the efficacy of following nutritional intervention. Moreover, WMT may improve clinical response to EN in patients who did not respond to nutritional therapy prior to WMT according to our previous report (5, 19). These studies confirmed that microbiota intervention contributed to response to nutritional treatment.

Besides, malnutrition might affect the efficacy of WMT. Hypoalbuminemia (defined by a serum albumin <35 g/L) may result in edema of intestinal mucosa among patients, which might affect the engraftment of microbiota. However, whether the albumin level could affect the efficacy of FMT is unsure. In the present study, the albumin level in group WMT-DAY1 at baseline was 33.8 ± 4.6 g/L and the minimum among all patients was 25.8 g/L, thus the current findings were based on a serum albumin level higher than 25 g/L. On the contrary, one study reported a cure rate of 91% after FMT(s) in patients with severe and severe-complicated CDI with a median albumin level of 25 g/L, among whom patients with lower albumin were more likely to undergo a repeat FMT (21).

Inflammation and active status are risk factors for developing malnutrition in patients with CD. In the present study, the CRP level tended to decrease at day 8 in both groups, indicating the anti-inflammation effect. Furthermore, lymphocyte count increased at day 8 and day 15 compared to that at day 1 in group WMT-DAY1, and the change of lymphocyte from day 1 to day 15 was significantly higher in group WMT-DAY1. Lymphocyte count could reflect both the nutritional and immunological status of the body and has been used as prognostic marker for complications when combining with serum albumin [known as prognostic nutritional index (PNI)] (22, 23). Gut microbiota was reported to have a pronounced modulatory effect on the immune system (24, 25). Our previous report showed that T lymphocyte, CD3+CD4+ cell and ratio of CD4+/CD8+ increased at 3 days post FMT (5). Therefore, we hypothesized that WMT could improve the homeostatic immune status. The lymphocyte count may rise after WMT as patient's nutritional and immune status improved at the same time. Our results indicated that patients in immediate WMT intervention group showed more increase in lymphocyte count. However, lymphocyte count did not increase at day 15 compared with that at day 1 in WMT-DAY8 group, which might be partially attributed to the small sample size. Hence, more evidence is needed to certify the role of WMT in lymphocyte count.

Prealbumin and albumin were considered as biochemical markers of nutritional status. Nowadays, they are considered as negative acute phase proteins and the decrease of which could reflect active CD (26). However, due to the short half-life of 2 days of prealbumin, it can reflect the early response to nutritional support (26). In the current study, the prealbumin increased significantly at day 8 and day 15 in group WMT-DAY1. An increase in prealbumin was also observed at day 8 and day 15 in group WMT-DAY8, but there was no statistical difference. At day 15, patients in both groups achieved significant increase in albumin. Though the decrease of inflammation might contribute to this change, the rise in prealbumin was not due solely to it but also nutritional support. The above results indicated that the strategy of EEN plus WMT might be superior to EEN alone. The WMT should be performed earlier for bringing more benefits to CD patients with malnutrition.

The result from a randomized trial demonstrated a remission rate of 53% in adult patients within 6 weeks of EEN (27). The pooled proportion of achieving clinical remission through FMT was 52% in patients with CD among cohort studies (28). The recent research reported FMT induced clinical response in 75.3% of patients (7). The current study reported a relative higher rate of clinical remission (87.5 and 72.7% at day 15 in group WMT-DAY1 and group WMT-DAY8 respectively), which might result from this combined treatment. In this study, immediate intervention of WMT seemed to show a trend of better clinical efficacy than delayed WMT intervention during 15 day's observation. However, no significant difference was identified, which might be attributed to the limited sample size.

The rate of WMT-related AE in CD was 4.26% according to the latest analysis based on the data from China Microbiota Transplantation System (CMTS) for the long-term evaluation on FMT safety (10). In the present study, four WMT-related AEs occurred within 1 month following WMT, which were mild and self-improved with no significant difference in the rate of AE between two groups. Therefore, the results demonstrated that immediate WMT intervention starting at day 1 improved efficiency of treating malnutrition and potentially did not increase the risk of AE.

There are some limitations to this study. The evidence from the current open-label, small sample sized study might not be convincing enough for making a conclusion. The observation period was short. Nutritional assessment needed to be performed in a more comprehensive way in a longer observation period. In addition, the lack of microbial analysis also prevented us from further understanding the mechanisms related to the changes in nutritional parameters as well as clinical activity. Further analysis of fecal microbiota among patients is warranted in the future study.

## Conclusion

This pilot randomized study provided preliminary evidence that EEN combined with immediate WMT intervention could rapidly improve the nutritional status and induce clinical remission in patients with CD complicated with malnutrition.

## Data Availability Statement

The original contributions presented in the study are included in the article/Supplementary Material, further inquiries can be directed to the corresponding author/s.

## Ethics Statement

The studies involving human participants were reviewed and approved by the Institutional Ethical Review Board of the Second Affiliated Hospital of Nanjing Medical University. The patients provided their written informed consent to participate in this study.

## Author Contributions

LX and YY: data curation, formal analysis, and writing—original draft preparation. FZ and YY: conceptualization. LX, YY, HZ, BC, and FZ: methodology. LX, YY, QW, and BC: investigation. LX, YY, XD, HZ, QW, BC, and FZ: writing—review and editing. BC: supervision. FZ: project administration. BC and FZ: funding acquisition. All authors have read and approved the final manuscript.

## Funding

This study was supported by the publicly donated Intestine Initiative Foundation; Jiangsu Provincial Medical Innovation Team (FZ); and National Natural Science Foundation of China (81670495, 81873548, and 81600417).

## Conflict of Interest

FZ conceived the concept of GenFMTer and transendoscopic enteral tubing and devices related to them. The remaining authors declare that the research was conducted in the absence of any commercial or financial relationships that could be construed as a potential conflict of interest.

## Publisher's Note

All claims expressed in this article are solely those of the authors and do not necessarily represent those of their affiliated organizations, or those of the publisher, the editors and the reviewers. Any product that may be evaluated in this article, or claim that may be made by its manufacturer, is not guaranteed or endorsed by the publisher.

## References

[1] Casanova MJ Chaparro M Molina B Merino O Batanero R Duenas-Sadornil C. Prevalence of malnutrition and nutritional characteristics of patients with inflammatory bowel disease. J Crohns Colitis. (2017) 11:1430–9. 10.1093/ecco-jcc/jjx102 28981652

[2] Massironi S Rossi RE Cavalcoli FA Della Valle S Fraquelli M Conte D. Nutritional deficiencies in inflammatory bowel disease: therapeutic approaches. Clin Nutr. (2013) 32:904–10. 10.1016/j.clnu.2013.03.020 23602613

[3] Ashton JJ Gavin J Beattie RM. Exclusive enteral nutrition in crohn's disease: evidence and practicalities. Clin Nutr. (2019) 38:80–9. 10.1016/j.clnu.2018.01.020 29398336

[4] Gatti S Galeazzi T Franceschini E Annibali R Albano V Verma AK. Effects of the exclusive enteral nutrition on the microbiota profile of patients with crohn's disease: a systematic review. Nutrients. (2017) 9:832. 10.3390/nu9080832 28777338 PMC5579625

[5] Cui B Feng Q Wang H Wang M Peng Z Li P. Fecal microbiota transplantation through mid-gut for refractory Crohn's disease: safety, feasibility, and efficacy trial results. J Gastroenterol Hepatol. (2015) 30:51–8. 10.1111/jgh.12727 25168749

[6] Sokol H Landman C Seksik P Berard L Montil M Nion-Larmurier I. Fecal microbiota transplantation to maintain remission in Crohn's disease: a pilot randomized controlled study. Microbiome. (2020) 8:12. 10.1186/s40168-020-0792-5 32014035 PMC6998149

[7] Xiang L Ding X Li Q Wu X Dai M Long C. Efficacy of faecal microbiota transplantation in Crohn's disease: a new target treatment? Microb Biotechnol. (2020) 13:760–9. 10.1111/1751-7915.13536 31958884 PMC7111085

[8] Zhang FM Wang HG Wang M Cui BT Fan ZN Ji GZ. Fecal microbiota transplantation for severe enterocolonic fistulizing Crohn's disease. World J Gastroenterol. (2013) 19:7213–6. 10.3748/wjg.v19.i41.7213 24222969 PMC3819561

[9] Fecal Microbiota Transplantation-standardization Study Group. Nanjing consensus on methodology of washed microbiota transplantation. Chin Med J. (2020) 133:2330–2. 10.1097/CM9.0000000000000954 32701590 PMC7546843

[10] Zhang T Lu G Zhao Z Liu Y Shen Q Li P. Washed microbiota transplantation vs. manual fecal microbiota transplantation: clinical findings, animal studies and in vitro screening. Protein Cell. (2020) 11:251–66. 10.1007/s13238-019-00684-8 31919742 PMC7093410

[11] Wang H Cui B Li Q Ding X Li P Zhang T. The safety of fecal microbiota transplantation for Crohn's disease: findings from a long-term study. Adv Ther. (2018) 35:1935–44. 10.1007/s12325-018-0800-3 30328062 PMC6223988

[12] Kondrup J Allison SP Elia M Vellas B Plauth M Educational and Clinical Practice Committee. ESPEN guidelines for nutrition screening 2002. Clin Nutr. (2003) 22:415–21. 10.1016/S0261-5614(03)00098-0 12880610

[13] Bauer J Capra S Ferguson M. Use of the scored patient-generated subjective global assessment (PG-SGA) as a nutrition assessment tool in patients with cancer. Eur J Clin Nutr. (2002) 56:779–85. 10.1038/sj.ejcn.1601412 12122555

[14] Long C Yu Y Cui B Jagessar SAR Zhang J Ji G. A novel quick transendoscopic enteral tubing in mid-gut: technique and training with video. BMC Gastroenterol. (2018) 18:37. 10.1186/s12876-018-0766-2 29534703 PMC5850973

[15] Ding X Li Q Li P Zhang T Cui B Ji G. Long-term safety and efficacy of fecal microbiota transplant in active ulcerative colitis. Drug Saf. (2019) 42:869–80. 10.1007/s40264-019-00809-2 30972640

[16] He Z Li P Zhu J Cui B Xu L Xiang J. Multiple fresh fecal microbiota transplants induces and maintains clinical remission in Crohn's disease complicated with inflammatory mass. Sci Rep. (2017) 7:4753. 10.1038/s41598-017-04984-z 28684845 PMC5500501

[17] Zhang F Cui B He X Nie Y Wu K Fan D. Microbiota transplantation: concept, methodology and strategy for its modernization. Protein Cell. (2018) 9:462–73. 10.1007/s13238-018-0541-8 29691757 PMC5960466

[18] Kelly CR Kunde SS Khoruts A. Guidance on preparing an investigational new drug application for fecal microbiota transplantation studies. Clin Gastroenterol Hepatol. (2014) 12:283–8. 10.1016/j.cgh.2013.09.060 24107393 PMC3947095

[19] Cui B Li P Xu L Peng Z Xiang J He Z. Step-up fecal microbiota transplantation (FMT) strategy. Gut Microbes. (2016) 7:323–8. 10.1080/19490976.2016.1151608 26939622 PMC4988439

[20] Griffin NW Ahern PP Cheng J Heath AC Ilkayeva O Newgard CB. Prior dietary practices and connections to a human gut microbial metacommunity alter responses to diet interventions. Cell Host Microbe. (2017) 21:84–96. 10.1016/j.chom.2016.12.006 28041931 PMC5234936

[21] Fischer M Sipe B Cheng YW Phelps E Rogers N Sagi S. Fecal microbiota transplant in severe and severe-complicated Clostridium difficile: A promising treatment approach. Gut Microbes. (2017) 8:289–302. 10.1080/19490976.2016.1273998 28001467 PMC5479393

[22] Buzby GP Mullen JL Matthews DC Hobbs CL Rosato EF. Prognostic nutritional index in gastrointestinal surgery. Am J Surg. (1980) 139:160–7. 10.1016/0002-9610(80)90246-9 7350839

[23] Feng Z Wen H Ju X Bi R Chen X Yang W. The preoperative prognostic nutritional index is a predictive and prognostic factor of high-grade serous ovarian cancer. BMC Cancer. (2018) 18:883. 10.1186/s12885-018-4732-8 30200903 PMC6131794

[24] Routy B Le Chatelier E Derosa L Duong CPM Alou MT Daillere R. Gut microbiome influences efficacy of PD-1-based immunotherapy against epithelial tumors. Science. (2018) 359:91–7. 10.1126/science.aan3706 29097494

[25] Wang Y Wiesnoski DH Helmink BA Gopalakrishnan V Choi K DuPont HL. Fecal microbiota transplantation for refractory immune checkpoint inhibitor-associated colitis. Nat Med. (2018) 24:1804–8. 10.1038/s41591-018-0238-9 30420754 PMC6322556

[26] Valentini L Schulzke JD. Mundane, yet challenging: the assessment of malnutrition in inflammatory bowel disease. Eur J Intern Med. (2011) 22:13–5. 10.1016/j.ejim.2010.07.021 21238886

[27] Lochs H Steinhardt HJ Klaus-Wentz B Zeitz M Vogelsang H Sommer H. Comparison of enteral nutrition and drug treatment in active Crohn's disease. results of the European cooperative Crohn's disease study IV. Gastroenterology. (1991) 101:881–8. 10.1016/0016-5085(91)90711-S 1679736

[28] Paramsothy S Paramsothy R Rubin DT Kamm MA Kaakoush NO Mitchell HM. Faecal microbiota transplantation for inflammatory bowel disease: a systematic review and meta-analysis. J Crohns Colitis. (2017) 11:1180–99. 10.1093/ecco-jcc/jjx063 28486648

