# Supplementary figures and images for: Exclusive Enteral Nutrition Plus Immediate vs. Delayed Washed Microbiota Transplantation in Crohn's Disease With Malnutrition: A Randomized Pilot Study
Source: Front Med (Lausanne). 2021 Oct 22;8:666062. doi: 10.3389/fmed.2021.666062 (PMC8569231; doi:10.3389/fmed.2021.666062)

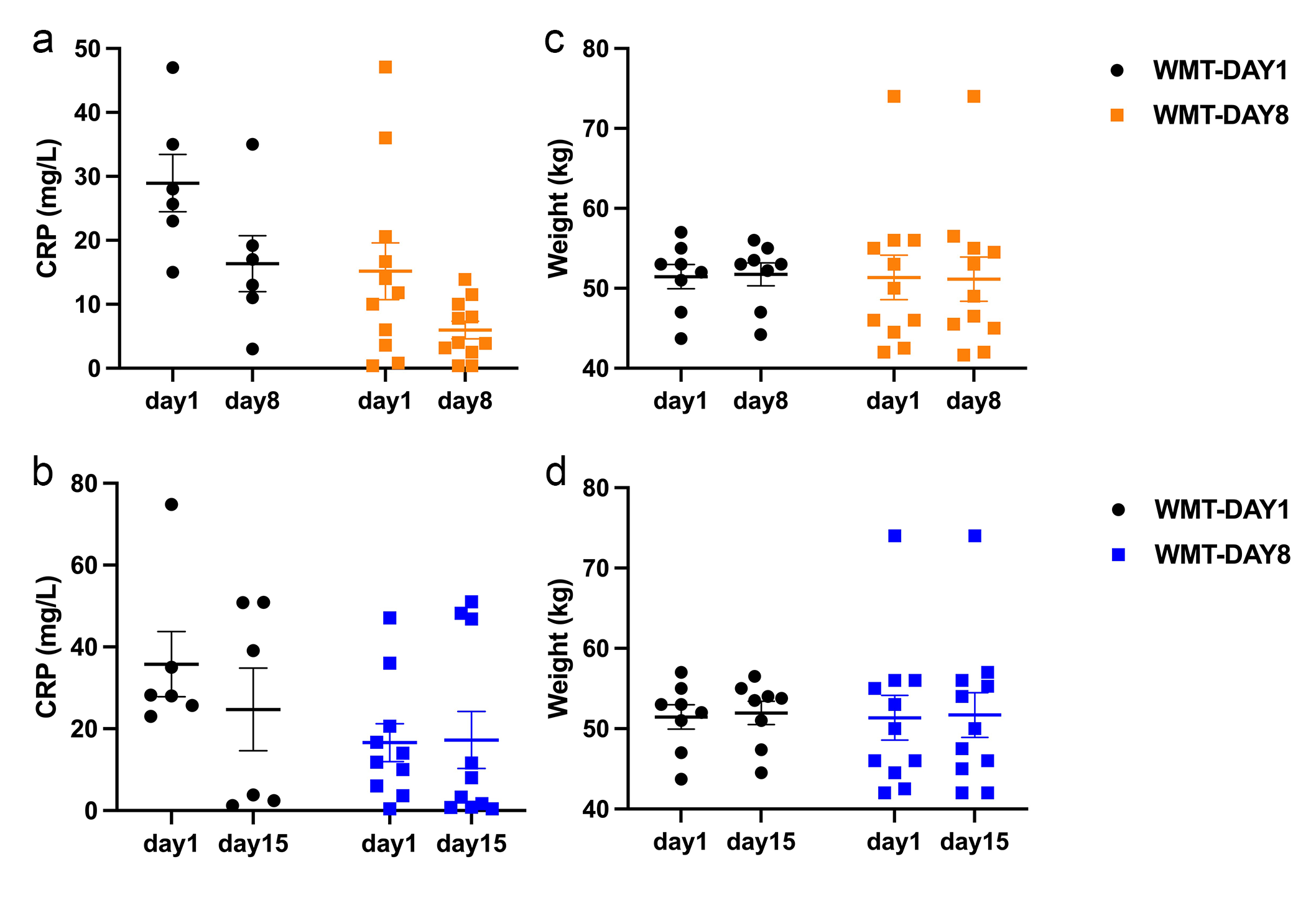

Supplement: Supplementary file 1 [file Image_1.TIF]
