# Supplementary material for: Exclusive Enteral Nutrition Plus Immediate vs. Delayed Washed Microbiota Transplantation in Crohn's Disease With Malnutrition: A Randomized Pilot Study
Source: Front Med (Lausanne). 2021 Oct 22;8:666062. doi: 10.3389/fmed.2021.666062 (PMC8569231; doi:10.3389/fmed.2021.666062)
Supplement: Supplementary file 2 [file Table_1.DOCX]

Figure S1. Changes in CRP and body weight between day 1 and day 8, day 1 and day 15 in two groups

a-b changes in CRP between day 1 and day 8, day 1 and day 15 in two groups. In group WMT-DAY1, data of two patients at day 8 and another two patients at day 15 were unavailable. In group WMT-DAY8, data of one patient at day 15 was unavailable. c-d changes in body weight between day 1 and day 8, day 1 and day 15 in two groups. The distribution of values within each group at each timing is illustrated by mean and SEM. CRP, C-reactive protein.
